# Account of Two Cases of Phthisis, Attended with Peculiar Circumstances

**Published:** 1834-01-01

**Authors:** Wm. Stroud

**Affiliations:** Physician to the Northern Dispensary, &c.


*Account of two Cases of Phthisis, attended with peculiar circumstances.* Communicated to the Harveian Society, by Wm. Stroud, m.d., Physician to the Northern Dispensary, &c.

Although phthisis has of late years been studied with much diligence, and success, the subject is by no means exhausted; and, on account of its frequency, and fatality, as well as of the valuable illustration which it affords to various branches of medical science, it is still a disease of great interest, and importance. Select cases, carefully observed, and faithfully reported, furnish, perhaps, the best mode of presenting medical facts in a clear, and natural point of view, and the best foundation for useful deduction, and theory. In prosecuting this object, insignificant details should, of course, be excluded; but some degree of prolixity, and minuteness is almost necessary, in order to place the reader as nearly as possible in the situation of the observer, and to satisfy him that the whole of the evidence, in its genuine, and original state, is submitted to his inspection. It should, moreover, be remembered that facts apparently minute frequently involve important principles; and that the suppression of circumstances seemingly inconsiderable, is often productive of serious obscurity, and misapprehension.

The two following cases of phthisis are distinguished by peculiar, and interesting features. In the first, the disease, which was confined to one lung, terminated in empyema, gangrene, and hemorrhage; in the second, while occupying one lung nearly in a crude state, it entirely destroyed the other, by converting it into an immense vomica, attended with obliteration of the pleural sac, and contraction of the side. These peculiarities, together with the mutual illustration of cases, remarkable alike for their similarity, and their diversity, and the conclusions naturally deducible from them, constitute their chief claim to attention.

*Case of Empyema of the left pleural sac, accompanied with tubercular, and gangrenous destruction of the subjacent lung.*

Towards the end of November, 1831, I was requested to visit, as a patient of the Northern Dispensary, Charles R*****d, aged thirty-one years, and formerly a merchant's clerk. I found him extremely ill, in the last stage of a pectoral disease, which he ascribed to cold; but, as one of his brothers was said to have died of phthisis, there was reason to suspect that he, also, possessed a similar constitution. More than two years before, being in the country, and returning home one night in a state of intoxication, while under the influence of mercury, used on account of a severe venereal disorder, he was bruised, and chilled by falling into a ditch; and, from that time, became the subject of the pulmonary complaint which ultimately terminated in his death. Soon after the commencement of his illness he made an excursion to Ireland, to try the effect of change of air, but returned worse than he went.

On the 12th of May, 1831, he became a patient of the Northern Dispensary, under the care of my colleague, Dr. Tweedie, who prescribed for him during a month; and having, by the use of the stethoscope, found pectoriloquy on the left side of the chest, but none on the right, and observing that, with little or no pain, he had hectic fever, a pulse of 120, and a troublesome cough, with purulent, and remarkably fetid expectoration, Dr. Tweedie regarded the case as advanced phthisis, and recommended him to live in the country, and to use a milk, and vegetable diet.

Shortly afterwards, and about five months before his death, a sudden, and peculiar sensation impressed him with the conviction that a pulmonary abscess had burst into the left pleural sac, in consequence of which the heart was pushed towards the right side, and, when the body was agitated, the fluctuation of a liquid could be distinctly heard on the opposite side. In compliance with Dr. Tweedie's advice, he ultimately went into the country, where for a while he seemed to improve; and, by his own suggestion, afterwards proceeded to France, with the intention of passing the winter in the southern part of that country; but, finding himself rapidly declining, he changed his mind, and returned to London. Yet, with the inconsistency, and self-delusion which are so characteristic of phthisical persons, he called on Dr. Tweedie, reported himself nearly well, and inquired whether he might be allowed to resume his former occupation as a merchant's clerk; a design from which he was, of course, prudently dissuaded. Shortly after his return to London, and about three weeks before his death, by violent efforts, of a character between vomiting, and coughing, he suddenly expectorated, in the course of three, or four days, five, or six pints of extremely fetid pus, unmixed with blood.

When I first saw him, a week later, (November 23, 1831,) his symptoms were as follows: On applying the stethoscope, the respiratory sound was observed to be loud, or puerile, throughout the whole of the right side of the chest, but inaudible throughout the whole of the left side, with the exception of a feeble murmur near the clavicle, and occasionally a low, and transient whisper in other parts. In like manner, the sound yielded by percussion was clear, and tympanic on the right side, but perfectly dull on the left; and from both indications it resulted that there was a large collection of liquid in the left pleural sac, without any mixture of gas. The patient, who had previously suffered pain in the sternum, and in the left side, was now free from pain, but lay better on the back than on either of the sides. He had a troublesome cough, attended with a sensation as if the left side of the chest were drawn inwards by a cord. His expectoration, which was thin, frothy, yellowish, and in considerable quantity, seemed to contain a portion of pus, and his breath was extremely fetid. He had some degree of hectic fever, much debility, and little sleep. Pulse in the recumbent posture 108, small, and weak. Tongue slightly white in the middle, urgent thirst, and scarcely any appetite. Bowels generally confined; urine copious, turbid, and dark-coloured. Having an intelligent, and highly sensitive mind, he described his symptoms with all that clearness, and precision which arises from a patient's long, and anxious study of his own complaint.

*Prescriptions.* Infus. Sennæ, f℥vi.; Aquæ Menth. vir. f℥ij.; Magnes. Sulphat. ℥ij. Sumatur f℥i. primo mane, prout opus fuerit.—Aquæ puræ, f℥vss.; Pulv. Acaciæ Gummi, ʒiv.; Sulphat. Quinin. gr. iv.; Acid. Sulphur. dilut. fʒi.; Tinct. Hyoscyam. fʒij.; Syrupi, fʒiv. Sumatur f℥i. ter. in dies.—Extract. Hyoscyam. ʒss.; Sulphat. Quinin. gr. iv.; Syrupi, q. s. Fiant pil. xij. quarum sumantur ij. omni vesp. horâ somni. Bibat, ad libitum, Decoct. Lichen. Island.

27th. He is rather weaker. His bowels have been relieved by the aperient mixture, but the pills fail to procure sleep. He is now unable to lie on the right side; and the left, or affected side of the chest is very tender, and painful on percussion. His breath is so offensive as to infect with a strong gangrenous odour, not only the chamber where he lies, but also the adjacent staircase; and the patient himself, being annoyed by it, frequently requests that one of the windows may be opened. Besides the general fetor, a volume of unmixed putrid gas seems occasionally to be discharged from the trachea.

*Prescriptions.* Repet. prout opus fuerit, Potio Cathartica, etiam Decoct. Lichen. Island. et Potio Tonica; sed, pro Sulph. Quinin. substituantur Tinct. Gent. comp. fʒiv.

December 1st. The symptoms are not relieved, but, on the contrary, the patient is weaker, with more of restlessness and anxiety, and seems to have some uneasiness about the left temple, which he frequently strikes with his hand. Thoroughly wearied, and distressed by the various annoyances attending his complaint, and, at the same time, hopeless of amendment, he expressed a wish that, by bleeding, or otherwise, his remaining strength may be rapidly exhausted, and his death accelerated; a wish which, as will presently be seen, was soon afterwards spontaneously accomplished.

*Prescriptions.* Pectori sinistro affigantur hirud. vi.—Sumat horâ somni, vel quoties opus fuerit, Ext. Hyoscyam. gr. v.—Aquæ puræ, f ℥vi.; Potass. Nitrat. ʒi.; Pulv. Acaciæ Gummi, ʒiv.; Tinct. Hyoscyam. f ʒij.; Tinct. Gent. comp. f ʒiij. Sumatur f ℥i. ter, vel quater in dies. Repet. si opus fuerit, Potio cathartica.

On the afternoon of this day, my respected colleague, Dr. Roget, joined me in visiting the patient; and, having duly considered the previous history, the stethoscopic indications, and the other symptoms, agreed in opinion that there was a large collection of liquid in the left pleural sac, and that, although the case seemed to be hopeless, temporary relief might, perhaps, be afforded by tapping. An opportunity for trying this operation never, however, occurred; for the following is the final report.

2d. The leeches drew a good deal of blood. During the past night, the patient suddenly expectorated two, or three pints of bloody liquid; after which he became rapidly exhausted, and, conscious of the approach of death, which he ardently desired, died tranquilly about two in the afternoon.

*Post-mortem Appearances.* The body was inspected by me on the 4th of December, nearly forty-eight hours after the patient's death, in the presence of Professor Carswell, Mr. Kiernan, Doctors Roget, Tweedie, and Theophilus Thompson, when the following appearances were observed.

*General Conditions.* The body was, in general, well-proportioned, and reduced, but not emaciated. It contained no fat, and little blood, except what was extravasated. Although, during the last days of life, its gangrenous odour had been almost intolerable, its fetor after death was inconsiderable. The countenance was pale, and placid, with some remains of coagulated blood about the nostrils, and mouth. The chest was rather narrow: its left, or diseased side was more prominent than the right, and livid from ecchymosis, especially at the middle, where the leeches had been applied.

The head and spine were not examined.

*Thorax.* On raising the front of the chest, the cavity of the left pleura was immediately exposed; its anterior half presenting the appearance of a vast, oblong, empty sac; its posterior, or decumbent half, being occupied by an opaque, black liquid; above which, towards the upper end, emerged a large, loose flake of equally black false membrane. The pleura was universally thickened by chronic inflammation, and fibrinous deposit; its surface being rough, dark-coloured, and speckled with short, angular, blackish lines, owing apparently to the engorgement of its minute veins, or absorbents. On the inner surface of the sternum, a median line distinctly divided this diseased pleura from the perfectly sound membrane of the opposite side.

After removing the flake of false membrane, and three or four pints of the black liquid in which it was immersed, and which seemed to be blood in a dissolved or altered state, the remains of the left lung, at length, came into view. It was a blackish, irregular mass, lying in the upper, and posterior part of the left side of the chest, and firmly adherent in several points to the costal pleura. When extracted, it was found to be much reduced in size; and, when washed, and cleansed from the inky liquid by which it had been inundated, its colour was dark blue. The upper portion of this lung was still tolerably sound, but interspersed with a few crude tubercles, varying from the size of a pea, to that of a small bean. The divided bronchia were rough, and somewhat dilated; and their lining membrane was thick, and stained by the black liquid. The lower portion was much broken, and excavated, and some of its fragments were gangrenous, and nearly detached. It contained several vomicæ, about the size of a large walnut, freely communicating with each other; and, by a number of round holes, some of which would admit a finger, opening into the pleural sac. When emptied of all its contents, this sac was found to be very extensive, and dilated. By the pressure of the confined liquid, the ribs had been somewhat protruded, the diaphragm depressed, and even the spinal column a little hollowed, the heart being, at the same time, slightly pushed towards the right side.

With the exception of the left pleura, and its contents, the body was substantially in a healthy state. The right lung merely presented a little œdema in its posterior, or decumbent portion, and a little reddish serum in its pleural sac; both probably the result of effusion occurring, either after death, or immediately before it. On making sections into this lung, it was found perfectly light and spongy, without a single tubercle, or any trace of inflammation. The heart, although so closely in contact with the diseased mass, was also quite sound, and nearly empty. The pericardium contained a small quantity of limpid watery liquid. The auriculæ propriæ were small, the walls of the left ventricle thick and strong, and the pulmonary artery rather dilated.

*Abdomen.* The omentum was retracted under the liver, and perfectly destitute of fat. The stomach was of moderate size. The intestines were externally of a chocolate colour; which, on opening them, was found to depend on a portion of the blood expectorated a little before death having been swallowed. This blood, in the state of broken coagulum, resembling dark currant jelly, could be traced as far as the cœcum, where it began to be mixed with feculent matter. There was no inflammation nor ulceration of the mucous membrane at the lower extremity of the ileum, nor any enlargement of the mesenteric glands. Owing, probably, to the violent expectorant efforts, the arch of the colon was empty, and much contracted; while the cœcum and the sigmoid flexure were somewhat dilated. The liver was large and protuberant, but healthy; its decumbent portion merely exhibiting the usual cadaveric discoloration. The gall-bladder contained a moderate quantity of thin, yellow bile, without concretions. The spleen was large, quadrangular, and of a pale pink colour, both internally, and also in distinct patches on its peritoneal coat. The kidneys were in like manner rather pale, but quite sound. The urinary bladder was considerably distended, owing either to the weakness of the patient in his last moments, or to his delicacy towards his mother, and another female, who latterly were almost always in attendance on him.

Remarks. This case presents a striking example of pulmonary consumption, confined to one lung, and varied in its character by the bursting of vomicæ into the pleural sac, followed by universal inflammation of that membrane, and by an accumulation, first of pus, and afterwards of blood, within its cavity. In some of the lower animals, tubercular deposits may be induced, almost at will, by certain modifications of food, exercise, or temperature; but, in the human race, the frequent failure, or absence of such causes, in conjunction with the evidence of family history, shows that a special predisposition, usually derived from parents, is more actively concerned in their production. In the present case, the influence of hereditary predisposition is by no means denied, more especially as one of the patient's brothers is reported to have died of phthisis; but it seems to have been comparatively feeble, for, with the exception of the left lung, no part of the body exhibited any trace of strumous affection, and the accidental causes which immediately excited the disease were of a powerful nature; namely, a violent chill, combined most likely with local violence, occurring in the night, and during the action of mercury employed for the cure of a severe venereal disorder. It is, indeed, remarkable that the right lung, and the heart, situated in the close vicinity of a part so thoroughly disorganized, and corrupted, should never have sustained the slightest injury; a circumstance which furnishes another proof of the weakness of the predisposition.

For a long time the tubercular disease of the left lung seems to have proceeded slowly, and in the usual manner, to the stage of softening and suppuration, according to the testimony of Dr. Tweedie, who, having examined the patient about six months before his death, observed pectoriloquy, with hectic fever and purulent expectoration, but no appearance of pleurisy, empyema, or pneumothorax. By the bursting of some of the more superficial vomicæ into the pleural sac, an event of which the patient was distinctly conscious, these conditions were, however, soon afterwards superadded. That the membrane became universally inflamed, was apparent from its thickened and coated state in the dead body; and, thenceforth, by the union of its own secretions with those of the ulcerated lung, several pints of purulent liquid, together with a portion of gas, were slowly collected in its cavity; as was proved by the splashing or fluctuating sound produced by agitation. The pressure resulting from this accumulation, which was sufficient to dilate in some degree the affected side of the chest, and to push the heart towards the other side, must, of course, have acted on the diseased lung; which, being free from adhesions, except near its summit, seems to have been gradually condensed, and impelled into the upper end of the pleural sac, where it was found after death. It was, probably, owing to this process, and to a valvular position of the perforated lung, like that of the ureters, more favorable to the descent of fluid than to its return, that such large collections took place, without any material interruption from expectoration. In the meanwhile, the increasing pressure naturally accelerated the progress of ulceration; and, by insulating and enfeebling some of the ulcerated fragments, induced gangrene, which was clearly announced by its peculiar and intolerable odour. The same pressure facilitated the exhalation of fetid air, both by ordinary expiration, and, occasionally, by sudden gusts; when, in consequence of some mechanical obstruction, portions of it had been longer detained, and had thereby become more offensive than usual. These gusts might, also, have been derived from the pus in the pleural sac, rendered putrescent by long confinement, and by partial communication with the outward air through the gangrened lung.

At length, by a violent effort of a character between vomiting and coughing, excited, perhaps, by the distention and irritation of the diaphragm on the affected side, the whole of this pus was rapidly expectorated, with temporary relief, but was soon succeeded by a similar collection of black and liquid blood; which might be ascribed partly to the sudden subtraction of internal pressure, while that of the atmosphere continued to operate, and partly to the usual tendency of pulmonary ulceration and gangrene to open neighbouring blood-vessels, and, more especially, some of the branches of the pulmonary artery. The amount of the two collections was nearly the same, but their circumstances were different. The first was of pus, derived chiefly from the inflamed pleura. Resisted by the reaction of the surrounding parts, it accumulated slowly during a period of about four months; and must at last have been entirely evacuated, since no pus was found in the sac after death. The second collection was of blood, supplied principally by the diseased lung. Encountering little resistance from parts already dilated, it took place more rapidly within a period not exceeding three weeks; and, owing to the exhaustion of strength thereby induced, the last evacuation was less complete than the former one, since between two and three pints only were expectorated, while a larger portion remained behind, amounting together to about six pints of blood; a quantity closely corresponding to that of the pus which had been previously discharged. The same excessive exhaustion naturally accounts for the patient's tranquil and easy death, which almost immediately ensued.

That the black liquid partly expectorated, and partly left behind in the pleural sac, and by which all the contiguous surfaces had been stained of an inky hue, was venous blood from the pulmonary artery, modified by the action of sulphuretted hydrogen gas, and perhaps also by other causes, there can be little doubt; since, on this supposition, the source of supply was close at hand, while none other can reasonably be imagined. It is a remarkable fact that, although the large quantity found in the pleural sac was perfectly black, and liquid, that which, in the process of evacuation, was left about the nostrils, and lips, or had descended into the stomach and bowels, was of a brighter colour, and partially coagulated. This change may, probably, be ascribed to the influence of atmospheric air, which is not only in contact with the external surface of the body, but of which, by means of a constant, and almost insensible deglutition, a considerable quantity seems to be always present in the alimentary canal.

It is also worthy of remark, that in this, as in many other instances of gangrene, the putrescent process was arrested by death; a result which may reasonably be attributed to the diminution thereby occasioned in the supply of air, heat, and blood to the affected parts; and, in some measure also, to the cessation of that more subtile influence, owing to which the dissolution of feeble parts is actively promoted by the superior vitality of stronger ones.

On the value, in a scientific point of view, of the physical signs derived from auscultation and percussion in cases like this, it is scarcely necessary to comment; since it is evident that, although the general history, when duly collected and considered, was highly significant, it would not have been sufficient, without the aid of these more precise indications, to ascertain the actual condition of the internal organs; namely, the perfectly healthy state of the right lung, the thoroughly disorganized state of the left lung, and the accumulation of a large quantity of liquid in the left pleural sac. The absence of pneumothorax, a little before the last evacuation, a circumstance not usual in cases of empyema, was, doubtless, occasioned by the peculiar situation of the internal parts, as previously explained; namely, by the reduced bulk and elevated position of the diseased lung, which allowed the confined liquid to accumulate until the air which might originally have been admitted was ultimately expelled through the same perforations by which it entered. This explanation is in exact accordance with the advanced period of the accumulation when the observation was made; for, had an examination taken place either before or after, it is most probable that the usual signs of pneumothorax, a clear resonance on percussion, accompanied by a total absence of respiratory murmur, would have been noticed.

On the treatment of so deplorable and desperate a disease, nothing satisfactory can be offered, since it is manifestly beyond the reach of all the ordinary resources of the healing art. The proposal to tap the left side of the chest was merely entertained from a desire to relieve, for the moment, the distress and anguish of the patient, occasioned by its distention; for, although, in idiopathic empyema, when the symptoms are urgent, and more especially when there is a tendency to spontaneous aperture, the operation may often be successful, in a secondary and complicated case, dependent on diseased lung, it must unquestionably be hopeless, and, generally speaking, inadmissible.

[The second case of modified Phthisis will be inserted in the ensuing Number.—Editor.]

